# Access to, use of, and experiences with social alarms in home-living people with dementia: results from the LIVE@Home.Path trial

**DOI:** 10.3389/fnagi.2023.1167616

**Published:** 2023-05-22

**Authors:** Nathalie Genevieve Søyland Puaschitz, Frode Fadnes Jacobsen, Line Iden Berge, Bettina Sandgathe Husebo

**Affiliations:** ^1^Faculty of Health Studies, VID Specialized University, Bergen, Norway; ^2^Department of Global Public Health and Primary Care, Faculty of Medicine, Center for Elderly and Nursing Home Medicine, University of Bergen, Bergen, Norway; ^3^Centre for Care Research West, Western Norway University of Applied Sciences, Bergen, Norway; ^4^Norske Kvinners Sanitetsforening (NKS) Olaviken Gerontopsychiatric Hospital, Askøy, Norway

**Keywords:** dementia, assistive technology, telecare devices, social alarm, indoor alarm, access, use, experience

## Abstract

**Background:**

Social alarms are considered an appropriate technology to ensure the safety and independence of older adults, but limited research has been conducted on their actual use. We, therefore, explored the access, experiences, and use of social alarms among home-bound people with dementia and their informal caregivers (dyads).

**Methods:**

From May 2019 to October 2021, the LIVE@Home.Path mixed-method intervention trial collected data from semi-quantitative questionnaires and qualitative interviews conducted among home-dwelling people with dementia and their informal caregivers in Norway. The study focused on data from the final assessment at 24 months.

**Results:**

A total of 278 dyads were included, and 82 participants reached the final assessment. The mean age of the patients was 83 years; 74.6% were female; 50% lived alone; and 58% had their child as a caregiver. A total of 62.2% of subjects had access to a social alarm. Caregivers were more likely to answer that the device was not in use (23.6%) compared to patients (14%). Qualitative data revealed that approximately 50% of the patients were not aware of having such an alarm. Regression analyses assessed that access to a social alarm was associated with increasing age (86–97 years, *p* = 0.005) and living alone (*p* < 0.001). Compared to their caregivers, people with dementia were more likely to answer that the device gave them a false sense of security (28% vs. 9.9%), while caregivers were more likely to answer that the social alarm was of no value (31.4% vs.14.0%). The number of social alarms installed increased from 39.5% at baseline to 68% at 24 months. The frequency of unused social alarms increased from 12 months (17.7%) to 24 months (23.5%), and patients were less likely to feel safe during this period (60.8% vs. 70%).

**Conclusion:**

Depending on their living situation, patients and family members experienced the installed social alarm differently. There is a gap between access to and the use of social alarms. The results indicate an urgent need for better routines in municipalities with regard to the provision and follow-up of existing social alarms. To meet the users’ changing needs and abilities, passive monitoring may help them adapt to declining cognitive abilities and increase their safety.

**Clinical Trial Registration**: https://ClinicalTrials.gov, NCT04043364.

## 1. Introduction

Dementia is a progressive, non-curable syndrome characterized by loss of cognitive functions such as memory, orientation, and language, changes in personality, and loss of the ability to perform everyday activities. More than 55 million people worldwide suffer from dementia, the majority of whom are women. Alzheimer’s disease is the most common form. As age and multimorbidity are the strongest known risk factors for dementia, the prevalence of dementia will increase sharply in the coming decades. In this aging population, dementia care management, service innovation, and caregiver support are the main targets to reduce the burden on the healthcare systems and their economic situation (Johannessen et al., 2019; World Health Organization, 2022).

In Norway, 40% of all people over 70 receiving home care suffer from dementia (Norwegian Directorate of Health, 2021). Municipalities have the primary responsibility for their care and for enabling them to live independently and safely at home for as long as possible (Thygesen, 2019). Informal care is provided by a considerable proportion of family caregivers (Hagen and Eide, 2020), making them indispensable collaborators with home care services (Oyebode et al., 2019). It is expected that traditional care services will not be sufficient in the future (Klimova et al., 2018; Nolte, 2018; Kristiansand Municipality, 2023).

At home, the installation and use of technological devices (i.e., telecare) may have the potential to support a person with dementia (PwD) and their formal and informal caregivers by improving resource use and service quality (Berge, 2017; Karlsen, 2019; Sriram et al., 2019; Vollmer and Ory, 2020). Telecare technology, such as a social alarm (SA), is often defined as safe and independent living technology that enables older adults to live longer and more safely in their own homes by addressing one of the key issues, such as the risk of falling accidents in the home or feelings of safety (Rantz et al., 2017; Johannessen et al., 2019; Thygesen, 2019). This type of technology can provide a sense of security for both the PwD and their caregiver. In this context, the SA is an active sensor that provides at-home, real-time, 24-h monitoring. The device typically consists of a body-worn pendant or wristband with a built-in alarm button that can be activated to summon help and a base unit that is connected to the PwD’s telephone line. When the alarm button is triggered, a signal is sent to a monitoring center or response center, where a trained operator speaks to the PwD through the base unit and assesses the situation. If necessary, the operator will contact home care services or ambulance services to respond to the emergency (Sjölinder and Avatare, 2014; Klimova et al., 2018).

In Norway, a SA is an indoor alarm that is usually offered by the municipalities, either for free installation or for a one-time installation fee of about 130 EUR. An additional monthly fee varies between approximately 25–35 EUR, depending on the municipality (Bergen Municipality, 2023; Kristiansand Municipality, 2023; Oslo Municipality, 2023). The fee includes monitoring and maintenance of the SA. In some municipalities, the SA is free of charge for low-income households (Oslo Municipality, 2023). Payment continues until the user cancels the service or dies. Additional sensors, such as a door sensor or smoke alarm, can be ordered for an additional one-time fee (Oslo Municipality, 2023).

In contrast to passive devices, the SA requires the user to have appropriate cognitive abilities, such as the ability to press the alarm button when needed (Cook et al., 2016). A Norwegian study concluded that the use of telecare, namely mobile social alarms, poses challenges that could lead to harm to elderly patients due to technological limitations and difficulties in understanding and managing the technology. To ensure their safety, it is important that telecare is closely followed up by homecare services and that the technology offered is adapted to each user’s individual abilities, skills, and resources (Johannessen et al., 2019). Most research on SAs describes either the use of or experiences with this technology (Sjölinder and Avatare, 2014; Korkmaz Yaylagul et al., 2022), but there are no known observational studies on the concrete use of the SA when installed at home, compared to the actual availability and experiences with it (Meiland et al., 2017). To justify the PwD’s monthly expenses for an installed SA, there is a need to question whether the municipalities have proper routines for the follow-up of existing SAs in order to ensure that the PwD can use this technology properly, when needed, and feel safe at home.

This study, therefore, investigates access to the SA compared to its use among home-living PwDs, together with the experiences of PwDs and their caregivers regarding this technology. We hypothesize that there is a gap between SA access and actual use.

## 2. Methods

The LIVE@Home.Path trial is a 2-year, stepped-wedge, cluster-randomized controlled trial involving home-living PwD and their informal caregiver (dyad) to investigate the efficacy of the multicomponent intervention on Learning, Innovation, Volunteerism, and Empowerment, which forms the acronym for the intervention. The principal scope of this article is the “Innovation” element, in terms of access to (here defined as “installed at home”) and experience with and use of a SA. This stepped-wedge trial used a closed-cohort design, meaning that all dyads were recruited before randomization (Hemming et al., 2018). All participants received a 6-month intervention during the 24-month trial, with the timing of the intervention determined by randomization. We used block randomization to allocate dyads to three intervention groups (Group 1, Group 2, and Group 3). The waiting intervention groups served as controls and received health care as usual.

The study was conducted between May 2019 and October 2021. Participants were blinded to allocation until their designated municipal coordinator contacted them to receive the LIVE intervention (Fæø et al., 2020). Before the coordinators evaluated enrolled patients for eligibility at baseline, they all took part in a 2-day course on the use of selected assessment tools, in addition to training on how to implement the intervention, including the provision of technical devices such as SAs. During the trial, a total of five cross-sectional data collections were conducted in each dyad’s home at baseline and at 6-, 12-, 18-, and 24-month data collection. For all dyads, the intervention was completed at the time of the 18-month data collection. The study protocol and more details about the study are described elsewhere (Husebo et al., 2020).

### 2.1. Study population

Between January and June 2019, dyads of home-bound men and women with dementia and their informal caregivers were screened for participation. They were recruited from memory clinics at local hospitals, municipal memory teams, and through advertisements in general media such as newspapers, radio, and TV. Dyads from three Norwegian municipalities were enrolled if the PwD met the following inclusion criteria: ≥65 years of age, diagnosed with dementia according to a standardized protocol, and a score of 15–26 on the Mini Mental State Examination (MMSE) (Arevalo-Rodriguez et al., 2015) or a score of 3–7 on the Functional Assessment Scaling Tool (FAST) (Sclan and Reisberg, 1992). The study population of interest in this study was stratified by access to a SA (yes/no) at 24 months.

### 2.2. Data description and analysis

This paper explored quantitative and qualitative data from the most recent data collection at 24 months. Supplementary qualitative data were added at 18 months. Data on demographics, medical history, and SAs were collected through semi-quantitative questionnaires in face-to-face interviews with the enrolled dyads. Questions about access to, use of, and experience with SAs were mainly collected through semi-quantitative questionnaires, initially directed at the PwD and then also answered by their caregiver immediately afterwards. Qualitative data from an 18- and 24-month data collection (19 and 7 interviews, respectively), were recorded and transcribed in order to highlight the quantitative data in the interpretation of the actual use and experience of the SA. Audio recordings were made during the interviews with the PwD and caregivers.

Supplementary data at baseline, 12-month, and 18-month data collection for access, use, and experience were analyzed for comparison. Data on experience and use from the baseline and 6-month data collection were not available.

#### 2.2.1. Primary outcomes

The primary outcome was access to, use of, and experience with SAs at the 24-month follow-up. These data were collected from both PwDs and caregivers via the following questions: (1) “*Do you have a social alarm?*,” and (2) “*If yes, what do you think about it?*” The first variable was dichotomous (yes/no), while the second variable was nominal and included seven different subcategories: not in use (yes/no), privately purchased (yes/no), safety (yes/no), false sense of safety (yes/no), more freedom (yes/no), time-consuming/burdening (yes/no), no value/no change (yes/no). Additional baseline, 12-month and 18-month data were evaluated.

#### 2.2.2. Covariates

Continuous variables were age (PwD and caregiver), number of diagnoses, and the following six validated assessment scores: Norwegian revised MMSE-NR3 (translated from the English version of the MMSE) (Norwegian National Advisory Unit on Ageing and Health, 2021), FAST (Sclan and Reisberg, 1992), Instrumental Activities of Daily Living (IADL) (Lawton and Brody, 1969), Personal Activities of Daily Living (PADL), Neuropsychiatric Inventory (NPI), and Cornell Scale for Depression in Dementia (CSDD)(Lawton, 1990).

Categorical variables regarding the PwD were investigated as follows: technology (yes/no), age in terciles (66–79/79–86/86–97 years), gender (male), living status (alone/with spouse/child or other), hazardous situations regarding falls (yes), type of dementia (Alzheimer’s disease/vascular dementia/Lewy-body dementia/frontotemporal dementia /mixed or unspecified dementia/other types), MMSE divided into three categories of dementia severity (normal/mild to moderate/severe), depression (yes/no), and anxiety (yes/no). The following categorical variables regarding caregivers were also explored: gender (male), relationship (sibling/child/friend/other), and their contribution to the care of the PwD in five categories (1–20%/21–40%/41–60%/61–80%/81–100%). Contribution to care was self-reported and depended on the total number of caregivers for each PwD.

### 2.3. Statistics

Descriptive data are reported as means (±SD) or as numbers (percentages) and are presented for the total population of PwDs at 24-month follow-up and stratified by access to SAs (no/yes). Differences between groups in access to SAs were tested with independent samples t-tests for normally distributed continuous variables, Mann–Whitney *U*-tests for non-normally distributed continuous variables, and chi-squared tests or Fisher’s exact tests for categorical variables. Adjusted multiple logistic regression analyses were used to investigate factors associated with access to a social alarm. Multivariate models were adjusted for age, gender, and cohabitation status. Covariates were selected based on our expertise from previous research on assistive technology and telecare in dementia care. Akaike’s information criterion guided model selection. Missing data were handled with listwise deletion. Calculations are expressed as odds ratios (OR) with 95% confidence intervals (CI), and *p*-values. Reported *p*-values <0.05 were considered statistically significant.

## 3. Results

### 3.1. Population

**Figure 1:**
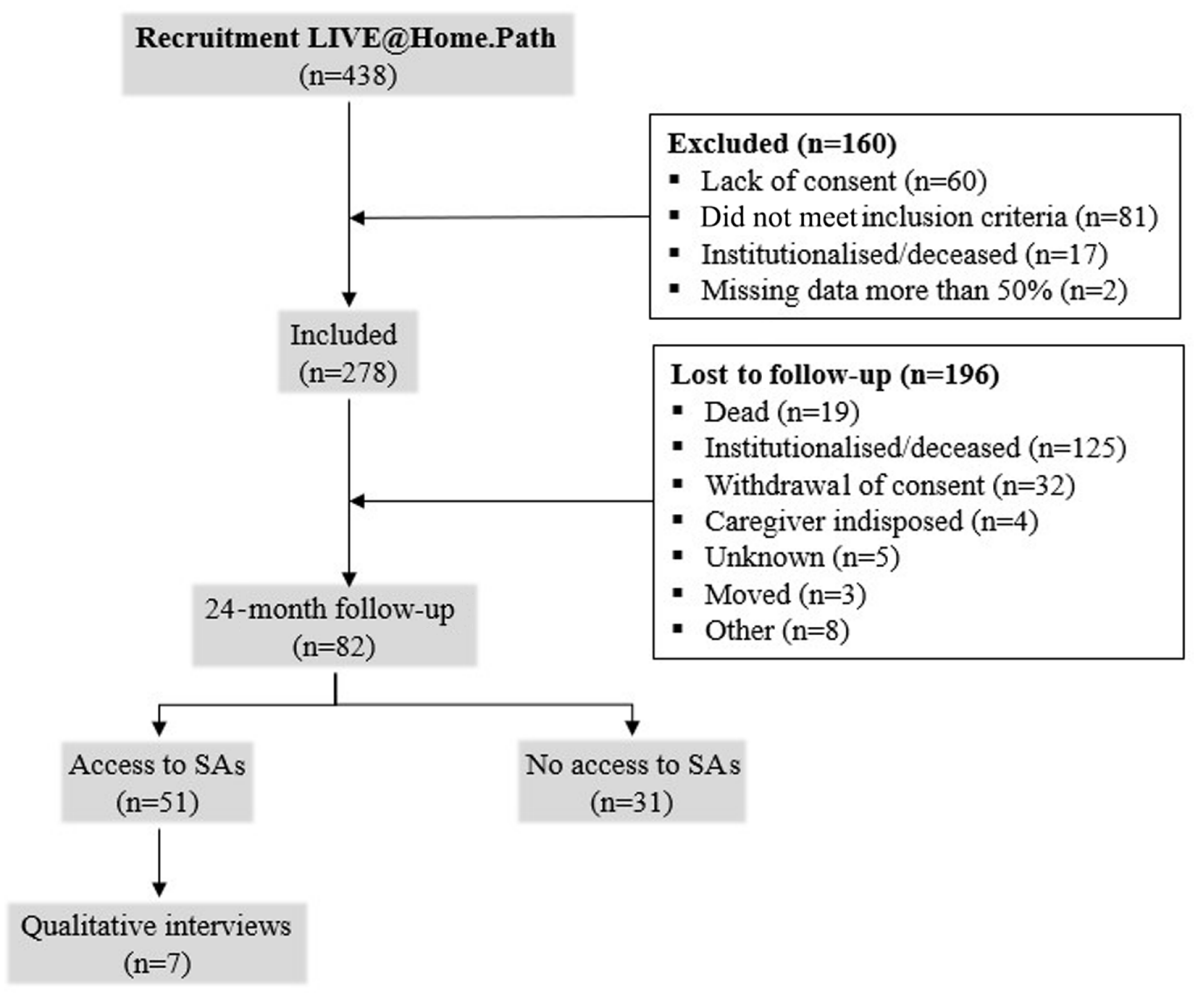
Flowchart of the study population. Illustrates the dyads of people with dementia (PwDs) and caregivers that were included and excluded. Available dyads for analysis at 24 months with and without installed social alarms (SA) and available qualitative interviews are shown. LIVE, Learning, Innovation, Volunteerism, Empowerment.

A total of 438 dyads of home-living PwDs and their informal caregivers were screened between January and June 2019, and 278 dyads were included at baseline. A total of 160 dyads were excluded due to lack of consent (*n* = 60), failure to meet inclusion criteria (*n* = 81), institutionalization or death (*n* = 17), and more than 50% missing data (*n* = 2). At the 24-month follow-up, 82 dyads were still participating in the trial. Reasons for dropping out (*n* = 196) were as follows: 19 PwDs had died, 125 had been institutionalized, 32 withdrew consent, 4 had an indisposed caregiver, 5 had unknown reasons, 3 had moved to another city, and 8 had other reasons for dropping out. Of the remaining dyads, 51 had access to a SA, and 7 were available for a qualitative interview (Figure 1).

Characteristics for the total population and groups with and without access to an SA at the 24-month data collection are presented in Table 1. The mean age of the total population of PwDs was 82.9 ± 6.9 years (range 66–97 years). The minority were men (35.4%), and 50.0% lived alone. Approximately 27% had experienced a fall accident at home. The mean MMSE-NR3 score was 17.9 ± 5.0, and the majority of PwDs had mild to moderate dementia (79.3%). The mean FAST score was 4.8 ± 1.0. The mean age of caregivers was 65.3 ± 12.3 years, with the majority being the children of the PwD (57.5%). Most caregivers (58.5%) contributed to more than 80% of the total care of the PwD.

**Table 1:** Characteristics of the total population at 24 months, and differences between people with dementia with and without an installed social alarm (SA).^1^

Characteristics	Total population *n* = 82	No SA *n* = 31 (37.8%)	SA *n* = 51 (62.2%)	*p*-value^2^
People with dementia				
Age	82.9 ± 6.9	76.4 ± 5.3	83.3 ± 6.3	<0.001
Age in terciles				<0.001
*66–79*	28 (34.2)	16 (51.6)	12 (23.5)	
*79–86*	27 (32.9)	13 (41.9)	14 (27.5)	
*86–97*	27 (32.9)	2 (6.5)	25 (49.0)	
Gender, male	29 (35.4)	17 (54.8)	12 (23.5)	0.004
Cohabitation status				<0.001
*Alone*	37 (50)	5 (16.7)	32 (72.7)	
*Spouse*	37 (50)	25 (83.3)	12 (27.3)	
*Child*	0 (0.0)	0 (0.0)	0 (0.0)	
*Other*	0 (0.0)	0 (0.0)	0 (0.0)	
Fall at home	22 (27.2)	3 (9.7)	19 (38.0)	0.005
Dementia etiology				0.01
*Alzheimer’s disease*	37 (45.1)	21 (67.7)	16 (31.4)	
*Vascular dementia*	2 (2.4)	0 (0.0)	2 (3.9)	
*Lewy- body dementia*	0 (0.0)	0 (0.0)	0 (0.0)	
*Frontotemporal dementia*	0 (0.0)	0 (0.0)	0 (0.0)	
*Unspecified dementia*	41 (50.0)	10 (32.3)	31 (60.8)	
*Dementia in other specified diseases*	2 (2.4)	0 (0.0)	2 (3.9)	
MMSE-NR3^3^	17.9 ± 5.0	17.5 ± 5.8	18.1 ± 4.5	0.61
MMSE by dementia severity categories^4^				0.02
*Normal*	9 (11.0)	4 (12.9)	5 (9.8)	
*Mild to moderate*	65 (79.3)	20 (64.5)	45 (88.2)	
*Severe*	6 (7.3)	5 (16.1)	1 (2.0)	
FAST^5^	4.8 ± 1.0	4.7 ± 1.1	4.8 ± 0.9	0.59
IADL^6^	23.2 ± 5.7	22.5 ± 6.3	23.3 ± 0.78	0.59
PADL^7^	11.7 ± 3.9	10.9 ± 3.9	12.2 ± 0.55	0.15
NPI-12 total score^8^	16.2 ± 17.9	18.4 ± 17.8	14.9 ± 17.9	0.39
*Depression*	50 (61.0)	19 (61.3)	31 (60.8)	1.00
*Anxiety*	48 (58.5)	18 (58.1)	30 (58.8)	1.00
CSDD total score^9^	5.12 ± 5.4	5.7 ± 5.3	4.8 ± 5.4	0.44
Informal caregiver				
Age	66.0 ± 12.4	71.4 ± 10.4	61.6 ± 11.9	<0.001
Gender, male	97 (35.4)	12 (38.7)	17 (33.3)	0.80
Relationship				<0.001
*Spouse*	118 (43.1)	23 (76.7)	9 (18.0)	
*Sibling*	1 (0.4)	0 (0.0)	0 (0.0)	
*Child*	142 (51.8)	6 (20.0)	40 (80.0)	
*Friend*	2 (0.7)	0 (0.0)	0 (0.0)	
*Other*	11 (4.0)	1 (3.3)	1 (2.0)	
Contribution to care				0.007
*1–20%*	12 (4.4)	2 (6.5)	0 (0.0)	
*21–40%*	26 (9.6)	2 (6.5)	8 (15.7)	
*41–60%*	45 (16.7)	0 (0.0)	8 (15.7)	
*61–80%*	48 (17.8)	3 (9.7)	11 (21.6)	
*81–100%*	139 (51.5)	24 (77.4)	24 (47.1)	

^1^Continuous variables are presented as means (± standard deviation) and categorical variables as numbers and percentages (%).

^2^Differences between groups of SA (yes/no) were tested with independent samples *t*-tests for normally distributed continuous variables, Mann–Whitney *U*-tests for non-normally distributed continuous variables, and chi-squared tests for categorical variables.

^3^MMSE-NR3, Norwegian Revised Mini Mental State Examination. Range 0–30, a higher score indicates more intact cognitive function, missing, *n* = 2.

^4^MMSE score divided into three categories according to dementia severity; normal: 25–30 score; mild to moderate: 11–24 score; severe: 0–10 score, missing, *n* = 2.

^5^FAST, Functional Assessment Scaling Tool. Range 1–7, a high score indicates a high severity of dementia.

^6^IADL, Instrumental Activities of Daily Living. Range 8–31, measures eight items for a proxy assessment of the use of telephone, shopping, finances, public transportation, and household use. A high score indicates poor functioning.

^7^PADL, Personal Activities of Daily Living. Range 6–30, measures six items 1–5 for a proxy assessment of personal activities such as toileting, grooming, dressing, transferring, and eating. A high score indicates poor functioning.

^8^NPI-12, Neuropsychiatric Inventory. Scales 0–144, psychotic subsyndrome (delusions and hallucinations); Scales 0–24, hyperactive behavior (agitation, euphoria, irritability, disinhibition, aberrant motor behavior); Scales 0–60, mood (depression, apathy, sleep disturbances, and appetite changes); Scales 0–48, each domain scales 0–12, with domain scores ≥4 indicating symptoms of clinical relevance.

^9^CSDD, Cornell Scale for Depression in Dementia. Ranges 0–38, with ≥8 indicating depressive symptoms of clinical relevance.

### 3.2. Access and use

At the 24-month data collection (Table 1), 62.2% of PwDs had access to a SA. PwDs with an installed SA were older (49% were 86–97 years old), more likely to be women (76.5%), lived alone (72.7%), more likely to have experienced a fall at home (38.0%), and more likely to have mild to moderate dementia (88.2%), compared to those without access. Their main caregivers were younger (61.6 ± 11.9 years vs. 71.4 ± 10.4 years), more often their children (80.0% vs. 20%), and less often contributing more than 80% to their care (47.1% vs. 77.4%).

Compared to PwDs with access at baseline (Supplementary Table 1), PwDs with access to a SA at the 24-month data collection were more likely to experience a fall at home (38.0% vs. 5.8% at baseline). They also scored higher on anxiety, according to the NPI (58.8% vs. 34.0% at baseline).

When the SA was already installed at baseline (Supplementary Table 2), PwDs with access to a SA at the 24-month data collection were older (differences: 6.3 ± 1.5 years, *p* < 0.001) and also more likely to suffer from anxiety (42.9%) compared to those without access to an SA at the 24-month data collection but not at baseline (72.4%), *p* = 0.004.

Multivariate regression analyses (Table 2) revealed that the age of PwDs was associated with access to a SA [OR (95% CI) = 1.23 (1.09–1.40), *p* = 0.003], in particular belonging to the older age group of 86–97 years [OR (95% CI) = 12.9 (2.19–75.9), *p* = 0.005], and living alone [OR (95%CI) = 10.5 (3.11–35.8), *p* < 0.001]. No association was observed between dementia severity and the MMSE-NR3.

**Table 2:** Factors associated with access to social alarms among 82 home-living people with dementia at 24 months.^1^

	OR	95% CI	*p*-value^2^
People with dementia			
Age	1.23	1.09–1.40	0.003
Age in terciles			
*66–79*	1.00		
*79–86*	1.58	0.45–5.58	0.71
*86–97*	12.9	2.19–75.9	0.005
Gender, male	1.01	0.26–3.88	0.99
Cohabitation status			
*Alone*	10.5	3.11–35.8	<0.001
*Spouse*	1.00		
Fall accident at home	1.06	0.16–7.08	0.95
Dementia etiology			
*Alzheimer’s disease*	1.00		
*Vascular dementia*	NA	NA	NA
*Unspecified dementia*	1.39	0.35–5.50	0.64
*Dementia in other specified diseases*	NA	NA	NA
MMSE-NR3^3^	0.99	0.86-1.14	0.91
IADL^4^	0.99	0.89-1.11	0.9
PADL^5^	1.20	0.93-1.53	0.18
NPI-12 total score^6^	0.96	0.91–1.01	0.14
*Depression*	0.55	0.15–2.10	0.38
*Anxiety*	0.91	0.23–3.57	0.89
CSDD total score^7^	0.88	0.74–1.04	0.13
Informal caregiver			
Age	0.95	0.89–1.01	0.13
Gender, male	0.61	0.09–4.30	0.62
Relationship			
*Spouse*	1.83	0.04–93.8	0.77
*Child*	1.00		
*Other*	NA	NA	NA
Contribution to care			
*1–20%*	NA	NA	NA
*21–40%*	1.00		
*41–60%*	NA	NA	NA
*61–80%*	0.51	0.15–25.0	0.58
*81–100%*	0.17	0.14–10.6	0.73

^1^CI, confidence interval; OR, odds ratio, NA, not available.

^2^Logistic regression analyses adjusted for age, gender, and cohabitation status.

^3^MMSE-NR3, Norwegian Revised Mini Mental State Examination. Range 0–30, higher scores indicate better cognitive function.

^4^IADL, Instrumental Activities of Daily Living. Range 8–31, measures eight items for a proxy assessment of the use of telephone, shopping, finances, public transportation, and household use. A high score indicates poor functioning.

^5^PADL, Personal Activities of Daily Living. Range 6–30, measures six items 1–5 for a proxy assessment of personal activities such as toileting, grooming, dressing, transferring, and eating. A high score indicates poor functioning.

^6^NPI-12, Neuropsychiatric Inventory. Scales 0–144, psychotic subsyndrome (delusions and hallucinations); Scales 0–24, hyperactive behavior (agitation, euphoria, irritability, disinhibition, aberrant motor behavior); Scales 0–60, mood (depression, apathy, sleep disturbances, and appetite changes); Scales 0–48, each domain scales 0–12, with domain scores ≥4 indicating symptoms of clinical relevance.

^7^CSDD, Cornell Scale for Depression in Dementia. Ranges 0–38, with ≥8 indicating depressive symptoms of clinical relevance.

Caregivers were more likely to answer than PwDs that the SA was not being used (23.5% compared to 13.7%) (Table 3). In the qualitative interviews conducted during the 18-month data collection (Table 4), 54% of PwDs were not aware of having access to a SA when asked. Their caregivers often clarified that a SA had been installed in the PwD’s home. Moreover, 46% of PwDs did not wear the device on their body during the interview. One of the caregivers (the wife of a PwD) was wearing the SA instead of the PwD, explaining that it made her feel safer in case her husband fell and needed help getting up. One caregiver reported that the PwD abused the SA by accidentally or unknowingly triggering it. Qualitative interviews with seven dyads at 24 months revealed similar results (data not shown in table). The following is an example of a conversation between the researcher, a PwD, and a caregiver during the 24-month data collection:

**Table 3:** Differences in the use and experience of social alarms in people with dementia and their caregivers at 24 months.^1^

	PwD	Cg	*p*-value
	*n* = 50 (61.0%)	*n* = 51 (62.2%)	
Use			
Not in use	7 (14.0%)	12 (23.5%)	<0.001
Experiences			
False sense of security	14 (28.0%)	5 (9.9%)	<0.001
Safety	31 (60.8%)	27 (52.9%)	<0.001
More freedom	2 (4.0%)	0 (0%)	NA
Time-consuming/burdening	0 (0%)	1 (2.0%)	NA
No change/no value	7 (14.0%)	16 (31.4%)	<0.001

^1^Categorical variables as numbers and percentages (%). Cg, caregiver; NA, not available; PwD, person with dementia. Differences between PwDs and caregivers were tested using chi-squared- and Fisher*’*s exact tests.

**Table 4:** Interpretation of the use of social alarms from 19 interviews with the person with dementia at the 18-month data collection.

Did not know that they had an SA	54%
Did not carry the SA on their body	46%
Used by caregiver	15%
Used for false alarms only	8%

Researcher: *“Do you have technical helping devices, such as a SA?”*

PwD: *“No.”*

Caregiver: *“Yes, you do!”*

PwD: *“Oh yes, but it has been a long time since I had it […] and where is it?”* (asking the spouse).

Caregiver: “*It is hanging from the violin*” (pointing to the living room wall that is decorated with a hanging violin).

Data obtained at all available surveys (baseline and at 12, 18, and 24 months), including access to, use of, and experience with the SA, are presented in Supplementary Table 3. At baseline, 109 (39.2%) of the PwDs had a SA installed in their home. According to the caregivers, access to an SA had increased from 39.2% at baseline to 49.1% at 12 months, 49.6% at 18 months, and 62.2% at 24 months. Data after 6 months were not available. The use of the SA decreased from 12 months (17.7% not using) to 24 months (23.5% not using). At the 18-month data collection, caregivers were also more likely to answer that the social alarm was not in use (24.6%), in comparison with answers provided by the PwD (18.0%).

### 3.3. Experiences

Dyads experienced the SA differently (Table 3). At 24 months, PwDs were more likely to answer that they experienced the SA as giving them a false sense of security (28.0%), compared to their caregiver (9.9%), and they felt more often that the SA contributed to security (PwD: 60.8% vs. caregiver: 52.9%). Caregivers experienced more often that the SA did not offer any value or change (31.4%), compared to the experiences of the PwDs (14.0%). PwDs who lived alone appreciated the SA more often for safety reasons than PwDs who lived with their spouse [OR (95% CI) = 3.21 (1.15–9.00), *p* = 0.03] (Table 5).

**Table 5:** Association between PwDs living alone compared to living with their spouse and their access to the SA and experiences at 24 months, divided by PwDs and caregiver responses.

	Answered by PwD *n* = 50 (61.0%)				Answered by caregiver *n* = 51 (62.7%)			
	n	OR	95% CI	*p*-value^1^	n	OR	95% CI	*p*-value^1^
Access	32	11.3	3.44–37.16	<0.001	32	9.79	2.95–32.5	<0.001
Use								
Not in use	6	3.17	0.34–29.64	0.31	7	1–11	0.28–4.45	0.88
Experiences								
False sense of security	11	4.66	0.92–23.5	0.06	5	NA	NA	NA
Safety	18	3.21	1.15–9.00	0.03	14	3.36	1.11–10.19	0.03
No change/no value	1	1.72	0.29–10.01	0.55	9	1.61	0.47–5.48	0.45

^1^*p*-values are calculated using logistic regression. Models adjusted for age.

At 24 months, 60.8% of PwDs felt less safe due to the SA compared to their experience at 12 months (70.0%). At the end of the study, caregivers were more likely to observe that the installed SA was of no value to them (31.4%), compared to their experience at 12 months (12.9%) (Supplementary Table 2).

## 4. Discussion

The primary aim was to investigate the current access to, use of, and experience with SAs by home-living people with disabilities and their caregivers in Norway. We found that the prevalence of an installed SA increased from 39.2% at baseline to 62.2% at the end of the study, after 24 months. Increasing age was associated with access to a SA, and PwDs and caregivers experienced SAs differently. Age and living status (alone or with a spouse) of the PwDs were the main factors associated with access to a SA, while dementia severity was not associated. Caregivers were more likely to report that the SA was not being used at 24 months. This finding was supported by the results of the qualitative interviews. Despite the increased number of installed SAs at 24 months, their use decreased even more during the study. PwDs and caregivers experienced the usefulness and safety of SAs differently, especially considering the living status of PwDs. Those PwDs who had access to a SA since the start of the study were older and suffered more often from anxiety at 2 years than those PwDs who did not have access at baseline. These findings are of key importance in order for the municipalities and homecare services to evaluate their current routines in providing and following up on existing SAs for cognitively impaired homebound people, both to ensure that PwDs can use this technology properly when needed and feel safe at home and to justify the monthly fees paid by PwDs to the municipalities for the installed technology.

### 4.1. Access and use

The municipalities included in this study are supposed to offer a SA to all persons >75 years of age or > 85 years of age, or upon request. As age is an additional associated factor for access to an SA (Puaschitz et al., 2021), this emphasizes the influence of municipalities in mapping and purchasing SAs for home-living PwDs.

At first glance, the substantial increase in access to SAs from 2019 (at baseline) to 2021 (at the end of the LIVE intervention) seems to reflect a successful intervention for the benefit of PwDs. However, when specifically asked about the actual use, about one in three PwDs with access reported not using it, while in interviews, less than half of them were aware of having it. In light of these results, the gap between access and use may highlight the complexity of dementia care and the use of active sensor technology over time. It is not enough to offer technology to these patients; the need and usability must also be thoroughly investigated before installation and consistently evaluated over time as the dementia disease progresses.

### 4.2. Passive versus active sensors

It has been questioned whether an active device such as a SA is an appropriate technology for a PwD (Sjölinder and Avatare, 2014; Puaschitz et al., 2021). In a Norwegian study, two focus group interviews were conducted with 10 female homecare professionals (nurses and occupational therapists) in two municipalities to investigate homecare professionals’ perceptions of safety, related to the use of telecare by older adults. The study revealed that many individuals diagnosed with dementia had great difficulties managing and understanding the functions of a mobile safety alarm. They were unable to push the emergency button, speak into the base unit, or understand this function (Johannessen et al., 2019). PwDs in our results mainly had mild to moderate levels of dementia. However, the findings of the earlier study are in line with the results from our study, which suggests that the aim of a scheme to install SAs for homebound people with dementia should be to increase the use of the device, not only ensure access. This can be underlined by our quantitative results, which exposed a high disparity between access to and the use of SAs, which was also confirmed by qualitative interviews revealing that more than half of PwDs with access to a SA was not aware of having one.

The advantage of passive sensor devices is that the user is passive while the technology is active, such as monitoring technology (Puaschitz et al., 2021). This type of technology has been suggested for PwDs because it does not require any cognitive abilities on the part of the PwD or the caregiver but also offers an individually tailored alert system that acts on specific movements, such as sitting up in bed, getting out of bed, or entering another room. This technology can also detect a fall if the user is lying on the floor. An alert is immediately sent to a monitoring center and/or to homecare staff by a mobile application (RoomMate, 2023). In fact, there is little research to confirm the appropriate use of this passive sensor technology in home-living PwDs.

### 4.3. Sustainability

Environmental sustainability has become a topic of widespread concern, and this also applies to healthcare (Richie, 2022). The reduction of the environmental footprint requires minimizing the production of technological devices such as SAs. Our results indicate that access to the SA is not an issue, but that only one in three PwDs actually uses their own. Therefore, there appears to be some potential for reducing the availability of SAs by evaluating the need for the SA in the first place, and by removing unused devices from the homes of PwDs.

### 4.4. Experience

The results of a study involving 19 qualitative interviews with PwDs in Norway suggest that PwDs have different experiences of telecare, such as SAs (Berge, 2017). This is reflected in our findings regarding PwDs’ experience of feeling safe. Our study also found that PwDs experienced the installed SA differently from their caregivers. This could be explained by the cognitive decline of PwD, resulting in their unawareness of the SA. Qualitative interviews revealed that, in one case, the caregiver (wife) wore the SA instead of the PwD. She explained that this made her feel safer in case her husband fell and needed help getting up (data not shown).

To enable older people to live at home for as long as possible, it is important that they feel safe in their homes. According to the results of previous studies, older subjects usually felt safe at home and had a better quality of life when a SA had been installed, knowing they would receive help if anything should happen (Porter, 2003, 2005; Barlow et al., 2007). In our study, PwDs with access to a SA felt less safe with the SA, especially if they lived together with their caregiver. This may be connected to the finding that these PwDs were also more likely to experience a fall accident at home. Moreover, our results showed that the longer the SA was installed in the home (from baseline to 24 months), the more often the PwD struggled with anxiety (Supplementary Table 3). This may indicate that social alarms are not an appropriate technology to prevent anxiety, which may potentially be connected to their increasing tendency to fall.

In Norway, SAs are offered to all people >75 years or > 85 years of age, or upon request, without the need for a proper evaluation (Puaschitz et al., 2021). Therefore, it could be argued that this device in particular is not chosen by the PwD themselves but by the caregiver or imposed by the municipalities. Since our results present a huge gap between access and use of SAs in PwDs, municipalities and their healthcare workers should have an increased focus on personalized healthcare through proper evaluations before offering a SA.

### 4.5. Strengths and limitations

The strengths of this study are the multicenter cohort, with data from more than 540 home-living male and female PwDs and their caregivers, including data collected at different points over the course of 2 years with information regarding access to, use of, and experience with an installed SA. The amount of available quantitative and qualitative data strengthens our results. The study cohort reflects the gender and age ratios of PwDs in the general population in Norway. The results may therefore be generalizable.

However, some limitations must be addressed. The data were mainly analyzed at the end of the study. Thus, it must be taken into account that the LIVE intervention may have positively influenced the data on access to SAs at 24 months.

Therefore, our results may not mirror the general number of SAs in home-living PwDs. However, briefly presented data from the 12- and 18-month data collections display similar results according to the gap between access to and use of SAs, which might support our findings at 24 months. We did not have quantitative data on actual requests for, use of, and experience with SAs at baseline or 6-month data collection. Thus, we have no information on whether the use of the SAs differed from access at baseline, just like at 24-month data collection. Our findings must be interpreted with caution, as the study intervention with a focused supply of telecare devices, such as SAs, may have influenced the results on general access to a SA. Notably, we only had little qualitative data at 24 months and therefore had to supplement our analyses with qualitative data from the 18-month data collection. However, the results were similar.

### 4.6. Conclusion

Our findings highlight that the installation of a SA for a home-living PwD does not automatically mean that it is in use and that PwDs often experience a false sense of security from the SA. It is important to note that, although the SA is designed to provide an added layer of safety and security for individuals with dementia, it should not be relied upon as the sole means of protection. Due to cognitive decline and the PwDs’ unawareness of having a SA, active sensor technology may not be appropriate for their abilities and may not always be able to make them feel safe at home. Regular check-ins and monitoring by a caregiver or family member are still essential. Homecare workers should also be aware that PwDs may experience the installed SAs differently than their caregivers and that, in some cases, the caregiver may benefit from having the SA on behalf of the PwD for their sense of safety. The results presented here may raise an ethical conflict for the municipalities regarding their desire to offer PwDs more security at home, and the challenge of adapting and following up on active sensor technology such as SAs that, over time, can be costly for the PwD and caregivers. Our findings, therefore, suggest that Norwegian municipalities could review their routines for offering SAs to PwDs without proper evaluation and establish proper routines for the follow-up of existing SA devices. Further studies could explore the use of passive sensor technology among PwDs and compare these devices with SAs, focusing on usability and safety for PwDs.

## Data availability statement

The datasets presented in this article are not readily available because data analyzed in the current study are due to ethical reasons only available on reasonable request. Requests to access the datasets should be directed to NP, nathalie.puaschitz@vid.no.

## Ethics statement

The studies involving human participants were reviewed and approved by the Regional Committee for Medical and Health Research Ethics, Norway (2019/385), the Norwegian Medicines Agency, and the Data Inspectorate. The patients/participants provided their written informed consent to participate in this study.

## Author contributions

NP wrote the main text of the manuscript, performed all analyses, prepared the figure and all tables, and had primary responsibility for final approval. NP and LB contributed to the data collection. NP, BH, FJ, and LB drafted and revised the manuscript. BH was the primary investigator for the LIVE@Home.Path-trial and applied for funding from the RCN. All authors contributed to the article and approved the submitted version.

## Funding

This trial was funded by the Research Council of Norway (RCN), www.forskningsradet.no (Sponsor’s Protocol Code: 273581). The funding granted the positions of NP and LB and financed this study.

## Conflict of interest

The authors declare that the research was conducted in the absence of any commercial or financial relationships that could be construed as a potential conflict of interest.

## Publisher’s note

All claims expressed in this article are solely those of the authors and do not necessarily represent those of their affiliated organizations, or those of the publisher, the editors and the reviewers. Any product that may be evaluated in this article, or claim that may be made by its manufacturer, is not guaranteed or endorsed by the publisher.

## Abbreviations

CG: Caregiver
CI: Confidence interval
CSDD: Cornell Scale for Depression in Dementia
FAST: Functional assessment staging tool
GHMR: General Medical Health Rating
IADL: Instrumental activities of daily living
LIVE: Learning, Innovation, Volunteerism, Empowerment
MMSE-NR3: Norwegian revised mini mental state examination
NA: Not available
NPI: Neuropsychiatric Inventory
OR: Odds ratio
PADL: Personal activities of daily living
PwD: Person with dementia
SA: Social alarm
SD: Standard deviation
