# Supplementary material for: Access to, use of, and experiences with social alarms in home-living people with dementia: results from the LIVE@Home.Path trial
Source: Front Aging Neurosci. 2023 May 22;15:1167616. doi: 10.3389/fnagi.2023.1167616 (PMC10239917; doi:10.3389/fnagi.2023.1167616)
Supplement: Supplementary file 1 [file Table_1.DOCX]

Supplementary Material

**Supplemental Table 1:** Characteristics of the total population at baseline, and differences between people with and without an installed social alarm.^1^

| **Characteristics** | **Baseline**  **n=278** | **No SA**  **n=167 (60.5%)** | **SA**  **n=109 (39.5%)** | **P-value^2^** |
| --- | --- | --- | --- | --- |
| **People with dementia:** |  |  |  |  |
| Age | 82.2 ± 7.0 | 79.5 ± 6.5 | 86 ± 5.6 | <0.001 |
| Age in terciles |  |  |  | <0.001 |
| *66-79* | 94 (33.8) | 81 (48.5) | 12 (11.0) |  |
| *79-86* | 92 (33.1) | 61 (36.5) | 31 (28.4) |  |
| *86-97* | 92 (33.1) | 25 (15.0) | 66 (60.6) |  |
| Gender, male | 103 (37.2) | 80 (47.9) | 23 (21.1) | <0.001 |
| Cohabitation status |  |  |  |  |
| *Alone* | 137 (49.3) | 163 (97.6) | 101 (92.7) | 0.06 |
| *Spouse* | 136 (48.9) | 2 (1.2) | 7 (6.4) |  |
| *Child* | 4 (1.4) | 2 (1.2) | 1 (0.9) |  |
| *Other* | 1 (0.4) |  |  |  |
| Fall accident at home | 16 (5.8) | 7 (4.2) | 9 (8.3) | 0.16 |
| Dementia etiology |  |  |  | 0.01 |
| *Alzheimer’s disease* | 101 (36.5) | 71 (42.5) | 30 (27.5) |  |
| *Vascular dementia* | 11 (4.0) | 7 (4.2) | 4 (3.7) |  |
| *Lewy- body dementia* | 3 (1.1) | 3 (1.8) | 0 (0.0) |  |
| *Frontotemporal dementia* | 1 (0.4) | 1 (0.6) | 0 (0.0) |  |
| *Unspecified dementia* | 153 (55.2) | 78 (46.7) | 74 (67.9) |  |
| *Dementia in other diseases classified* | 1 (0.4) | 7 (4.2) | 1 (0.9) |  |
| MMSE-NR3^3^ | 20.4 ± 4.08 | 167 ± 0.32 | 109 ± 0.39 | 0.32 |
| MMSE by categories of dementia severity^4^ |  |  |  | 0.36 |
| *Normal* | 40 (14.4) | 13 (17.1) | 27 (13.4) |  |
| *Mild-moderate* | 234 (84.2) | 63 (82.9) | 171 (84.7) |  |
| *Severe* | 4 (1.4) | 0 (0.0) | 4 (2.0) |  |
| FAST^5^ | 4.2 ± 0.9 | 4.1 ± 0.96 | 4.3 ± 0.77 | 0.01 |
| IADL^6^ | 19.9 ± 6.1 | 19.3 ± 6.3 | 20.8 ± 5.7 | 0.05 |
| PADL^7^ | 10.3 ± 3.3 | 9.8 ± 3.3 | 11.1 ± 3.2 | 0.001 |
| NPI-12 total score^8^ | 16.7 ± 15.7 | 15.2 (14.0) | 19.1 ± 17.8 | 0.12 |
| *Depression* | 146 (52.5) | 59 (36.4) | 61 (59.2) | <0.001 |
| *Anxiety* | 167 (60.1) | 63 (39.1) | 35 (34.0) | 0.40 |
| CSDD total score^9^ | 5.8 ± 4.9 | 5.6 ± 4.7 | 6.2 ± 5.3 | 0.47 |
| **Informal caregiver:** |  |  |  |  |
| Age | 66.0 ± 12.4 | 68.3 ± 12.9 | 62.5 ± 10.7 | 0.02 |
| Gender, male | 97 (35.4) | 58 (34.9) | 38 (35.9) | 0.02 |
| Kinship |  |  |  | <0.001 |
| *Spouse* | 118 (43.1) | 100 (62.2) | 18 (17.0) |  |
| *Sibling* | 1 (0.4) | 1 (0.6) | 0 (0.0) |  |
| *Child* | 142 (51.8) | 58 (34.9) | 82 (77.4) |  |
| *Friend* | 2 (0.7) | 1 (0.6) | 1 (0.9) |  |
| *Other* | 11 (4.0) | 6 (3.6) | 5 (4.7) |  |
| Care contribution |  |  |  | <0.001 |
| *1-20%* | 12 (4.4) | 4 (2.4) | 8 (7.7) |  |
| *21-40%* | 26 (9.6) | 1 (6.7) | 15 (14.4) |  |
| *41-60%* | 45 (16.7) | 21 (12.7) | 23 (22.1) |  |
| *61-80%* | 48 (17.8) | 26 (15.8) | 22 (21.2) |  |
| *81-100%* | 139 (51.5) | 103 (62.4) | 36 (34.6) |  |

^1^ Continuous variables are presented as mean values (± standard deviation) and categorical variables as numbers and percentages (%).

^2^ Differences between groups of SA (yes/no) were tested with independent samples t tests for normally distributed continuous variables, Mann-Whitney U-tests for nonnormally distributed continuous variables, and Chi-squared tests for categorical variables.

^3^ MMSE-NR3, Norwegian revised Mini Mental State Examination. Range 0-30, a higher score indicates more intact cognitive function.

^4^ MMSE score divided into three categories according to dementia severity; normal: 25-30 score; mild-moderate: 11-24 score; severe: 0-10 score.

^5^ FAST, functional assessment scaling tool. Range 1-7, a high score indicates a high severity of dementia.

^6^ IADL, instrumental activities of daily living. Range 8-31, measures 8 items for proxy assessment of use of telephone, shopping, economy, public transport, and household; a high score indicates poor function.

^7^ PADL, personal activities of daily living. Range 6-30, measures 6 items 1-5 for proxy assessment of personal activities such as toileting, grooming, dressing, transfer, and eating. A high score indicates poor function.

^8^ NPI-12, Neuropsychiatric Inventory. Ranges 0–144, psychosis subsyndrome (delusions and hallucinations) ranges 0–24, hyperactive behavior (agitation, euphoria, irritation, disinhibition, aberrant motor behavior) ranges 0–60, mood (depression, apathy, sleep disturbances, and appetite changes) ranges 0–48, each domain ranges 0–12 with domain scores ≥4 indicating symptoms of clinical relevance.

^9^ CSDD, Cornell Scale for Depression in Dementia. Ranges 0–38, ≥8 indicate depressive symptoms of clinical relevance.

**Supplemental Table 2**: Access, use and experiences of the social alarm at all available data collections, answered by people with dementia and their caregivers.^1^

|  |  | **Month of data collection** | | |
| --- | --- | --- | --- | --- |
|  | Baseline  n=278 | 12  N=171 | 18  N=123 | 24  N=82 |
| **Access:** |  |  |  |  |
| Answer from the CG | 109 (39.2) | 84 (49.1) | 61 (49.6) | 51 (62.2) |
| Answer from the PwD |  | 87 (50.9) | 65 (47.2) | 50 (61.0) |
| **Use:** |  |  |  |  |
| Not in use (CG) | NA | 15 (17.7) | 15 (24.6) | 12 (23.5) |
| Not in use (PwD) | NA | 10 (11.9) | 11 (18.0) | 7 (13.7) |
| **Experiences:** |  |  |  |  |
| Safety (CG) | NA | 56 (65.9) | 37 (60.7) | 27 (52.9) |
| Safety (PwD) | NA | 61 (70.0) | 42 (64.6) | 31 (60.8) |
| False sense of safety (CG) | NA | 5 (5.9) | 11 (18.0) | 5 (9.9) |
| False sense of safety (PwD) | NA | 18 (20.7) | 19 (29.2) | 14 (28.0) |
| More freedom (CG) | NA | 0 (0) | 0 (0) | 0 (0) |
| More freedom (PwD) | NA | 3 (3.6) | 0 (0) | 2 (4.0) |
| Time-consuming/burdening (CG) | NA | 1 (1.2) | 2 (3.3) | 1 (2.0) |
| Time-consuming/burdening (PwD) | NA | 2 (1.7) | 0 (0) | 0 (0) |
| No change/no value (CG) | NA | 11 (12.9) | 12 (19.7) | 16 (31.4) |
| No change/no value (PwD) | NA | 5 (6.0) | 4 (6.2) | 7 (8.5) |

^1^ Categorical variables as numbers and percentages (%). Abbreviations: CG, caregiver; PwD, person with dementia; NA, not available.

**Supplemental Table 3**: Characteristics and experiences for PwD and CGs with access to SA at 24-months, stratified by with and without access to SA at baseline.^1^

|  | **PwD** | | | **Caregivers** | | |
| --- | --- | --- | --- | --- | --- | --- |
|  | **SA at baseline** | | | **SA at baseline** | | |
|  | **No**  **N=21 (42.0)** | **Yes**  **N=29 (58.0)** | **P-value** | **No**  **N=20 (39.2)** | **Yes**  **N=31 (60.8)** | **P-value** |
| **Characteristics** |  |  |  |  |  |  |
| Age | 81.9 ± 4.6 | 88.2 ± 6.1 | <0.001 | 81.9 ± 4.7 | 88.0 ± 6.2 | <0.001 |
| Gender (male) | 6 (28.6) | 4 (13.8) | 0.20 | 6 (30.0) | 6 (19.4) | 0.38 |
| Cohabitation (alone) | 14 (66.7) | 25 (86.2) | 0.10 | 13 (65.0) | 26 (83.9) | 0.12 |
| Fall | 6 (28.6) | 12 (41.4) | 0.87 | 5 (25.0) | 14 (45.2) | 0.15 |
| Alzheimer`s disease | 10 (47.6) | 7 (24.1) | 0.08 | 9 (45.0) | 7 (22.6) | 0.09 |
| MMSE^2^ | 17.2 ± 4.7 | 19.0 ± 4.2 | 0.17 | 17.2 ± 4.7 | 18.7 ± 4.3 | 0.24 |
| FAST^3^ | 4.7 ± 0.91 | 4.9 ± 0.89 | 0.47 | 4.6 ± 0.9 | 4.9 ± 0.9 | 0.20 |
| Depression | 13 (61.9) | 18 (62.1) | 0.99 | 13 (65.0) | 18 (58.1) | 0.62 |
| Anxiety | 9 (42.9) | 21 (72.4) | 0.04 | 9 (45.0) | 21 (67.7) | 0.11 |
| Caregiver age | 61.7 ± 13.0 | 60.9 ± 11.2 | 0.80 | 62.1 ± 13.2 | 61.3 (11.2) | 0.80 |
| **Experiences:** |  |  |  |  |  |  |
| Not in use | 1 (4.8) | 6 (20.7) | 0.11 | 5 (25.0) | 7 (22.6) | 0.84 |
| False sense of safety | 6 (28.6) | 8 (27.6) | 0.94 | 3 (60.0) | 2 (40.0) | 0.32 |
| Safety | 14 (66.7) | 18 (62.1) | 0.74 | 8 (40.0) | 19 (61.3) | 0.14 |
| More freedom | 0 (0) | 2 (6.9) | 0.22 | 0 (0) | 0 (0) |  |
| Time-consuming /burdening | 0 (0) | 0 (0) |  | 0 (0.0) | 1 (3.2) | 0.42 |
| No change/no value | 3 (14.3) | 4 (13.8) | 0.96 | 8 (40.0) | 8 (25.8) | 0.29 |

^1^ Categorical variables as numbers and percentages (%). Abbreviations: Cg, caregiver; PwD, person with dementia; SA, social alarm. Differences of experiences of the SA between PwDs and caregivers were tested by chi squared- and Fisher`s exact test.

^2^ MMSE-NR3, Norwegian revised Mini Mental State Examination. Range 0-30, a higher score indicates more intact cognitive function.

^3^ FAST, functional assessment scaling tool. Range 1-7, a high score indicates a high severity of dementia.
